# One-Year Outcome of Intravitreal Injection of Ranibizumab Biosimilar for Myopic Choroidal Neovascularization in Japanese Patients

**DOI:** 10.3390/jcm13164641

**Published:** 2024-08-08

**Authors:** Mami Tomita, Manabu Yamamoto, Kumiko Hirayama, Akika Kyo, Norihiko Misawa, Gen Kinari, Takeya Kohno, Shigeru Honda

**Affiliations:** Department of Ophthalmology and Visual Science, Graduate School of Medicine, Osaka Metropolitan University, Osaka 545-8585, Japan; mami.dnc21@gmail.com (M.T.); kumiko.hirayama126@hotmail.co.jp (K.H.); mingxiang.ac@gmail.com (A.K.); sannjaku@gmail.com (N.M.); k56rin@gmail.com (G.K.); takeyakohno@msn.com (T.K.); shonda@omu.ac.jp (S.H.)

**Keywords:** myopia, ranibizumab, choroidal neovascularization, anti-vascular endothelial agent

## Abstract

**Objectives:** To evaluate the one-year outcomes of intravitreal ranibizumab biosimilar (RBZ-BS) injections for myopic choroidal neovascularization (mCNV) in Japanese patients. **Methods:** Twenty-one patients (mean age 69.0 years; 4 males, 17 females) with high myopia and mCNV were retrospectively reviewed. Twelve were treatment-naïve, and nine had previous anti-VEGF treatments. Efficacy measures included best-corrected visual acuity (BCVA) and central macular thickness (CMT). **Results:** The treatment-naïve group showed significant BCVA improvement from 0.55 ± 0.34 at baseline to 0.24 ± 0.28 at 12 months. The previously treated group had no significant BCVA changes. CMT significantly decreased in both groups: from 295.3 ± 105.2 µm to 207.3 ± 63.0 µm in the treatment-naïve group, and from 196.1 ± 62.0 µm to 147.2 ± 50.1 µm in the previously treated group. Dry macula rates were high: 83% at 3 months and 83% at 12 months in the treatment-naïve group, and 67% at 3 months and 89% at 12 months in the previously treated group. No adverse events were reported. **Conclusions:** These findings indicate that RBZ-BS is an effective and safe treatment for mCNV, particularly in treatment-naïve patients. The use of RBZ-BS offers a cost-effective alternative to original ranibizumab, reducing financial burdens while maintaining high therapeutic efficacy. Further studies with larger sample sizes and longer follow-up periods are needed to confirm these results and evaluate long-term outcomes and cost-effectiveness.

## 1. Introduction

Pathological myopia is caused by excessive elongation of the eyeball, which leads to various degenerative changes in the retina and consequent loss of vision [1]. Myopic choroidal neovascularization (mCNV) is one of the leading causes of visual impairment associated with pathological myopia [1,2,3,4,5]. mCNV occurs when abnormal choroidal neovascularization penetrates the upper layers of the choroid, causing hemorrhage and exudation. Without intervention, this condition can severely affect central vision, significantly impacting patients’ quality of life [6]. The prevalence of pathological myopia is increasing worldwide, particularly in East Asia, making it a growing public health concern. This has spurred research and innovation in treatment approaches to manage and mitigate its effects.

The advent of anti-vascular endothelial growth factor (VEGF) drugs has marked a significant breakthrough in the treatment of mCNV. VEGF is a factor that stimulates the formation of neovascularization, and by inhibiting this process, the growth of these new blood vessels can be suppressed [7,8]. Ranibizumab, a monoclonal antibody that specifically inhibits VEGF, has been proven effective and safe in numerous clinical trials for conditions such as neovascular age-related macular degeneration (nAMD), diabetic macular edema (DME), and mCNV [9,10,11,12]. Its administration has shown substantial improvements in visual acuity and a reduction in disease progression. However, the high cost of ranibizumab remains a challenge, especially for long-term treatment, posing a financial burden on patients and healthcare systems.

In response to the financial challenges associated with the use of original biologic drugs, biosimilars have emerged as promising alternatives. Biosimilars are highly similar to original biologic drugs, requiring equivalent quality, safety, and efficacy [13,14,15,16,17,18]. They offer the potential for significant cost savings while providing comparable therapeutic outcomes. Notably, the newly launched ranibizumab biosimilar (RBZ-BS, Senju Pharmaceutical Co., Ltd., Osaka, Japan, in collaboration with Kidswell Bio Corporation, Tokyo, Japan) was approved in Japan in 2021 and is recognized as the first biosimilar product in the ophthalmology field in Japan [18,19]. This approval has paved the way for more affordable treatment options, potentially increasing accessibility for patients who require long-term therapy.

The introduction of biosimilars holds significant economic implications. As the global population ages, increasing medical costs have become a societal issue in Japan and other countries. In this context, biosimilars, which maintain high therapeutic efficacy while reducing treatment costs, play a crucial role in health economics [19,20,21,22]. They not only alleviate the financial strain on healthcare systems but also ensure that more patients can benefit from essential treatments without compromising on the quality of care.

Previous reports have demonstrated the efficacy of ranibizumab biosimilars for conditions such as nAMD and DME, but data on their use for mCNV are limited [18]. This lack of comprehensive data highlights the need for further investigation to fully understand the potential benefits and safety profiles of these biosimilars in treating mCNV. The objective of this study is to retrospectively evaluate the one-year treatment outcomes of intravitreal injections of RBZ-BS for mCNV, assessing its efficacy and safety.

## 2. Materials and Methods

### 2.1. Study Participants

We retrospectively reviewed the clinical charts of all patients initiated with intravitreal RBZ-BS (IVRBS) for mCNV at the Department of Ophthalmology of Osaka Metropolitan University Hospital between March 2022 and July 2022. The study adhered to the tenets of the Declaration of Helsinki and was approved by Ethical Committee of Osaka Metropolitan University Graduate School of Medicine (No. 2019-062, approval date, 16 December 2019), and written informed consent was obtained from all patients prior to treatment.

All patients were examined at the initial visit with best corrected visual acuity (BCVA) using a Landolt C chart, fundus examination with slit-lamp microscopy, fluorescein and indocyanine green angiography (FA, IA), optical coherence tomography (OCT), and multimodal imaging to diagnose mCNV. OCT angiography (OCTA) was also used for diagnosis when neovascularization was suspected and undetectable by other examinations. FA, IA, OCT, and OCTA were performed using confocal scanning laser ophthalmoscopy (HRA/Spectralis; Heidelberg Engineering Heidelberg, Germany).

### 2.2. Inclusion and Exclusion Criteria

The inclusion criteria for this study were as follows:

- Patients with subjective symptoms such as central scotoma, metamorphopsia, and reduced visual acuity due to mCNV;
- CNV present in the subfovea;
- Patient meets the diagnostic criteria for high myopia, defined as an ocular axis length of 26 mm or more or an equivalent spherical diameter of −6.0 D or more [5];
- Patients who had been treated with IVRBS for at least one year after initiation of treatment.

Those who did not meet the above criteria were all excluded from this study.

### 2.3. Treatment Methods

Intravitreal injections were carried out using the same standard procedure in all patients [23]. Each injection was performed under sterile conditions using a 30-gauge needle, with patients receiving topical anesthesia prior to the injection. The dosing of intravitreal RBZ-BS was administered on a monthly pro re nata (PRN) regimen following the initial dose of RBZ-BS. A dry macula was defined as the absence of subretinal hyperreflective exudation, all intraretinal and subretinal fluid, and intraretinal and subretinal hemorrhages, as assessed by optical coherence tomography (OCT). During each follow-up period, if a dry macula was not achieved, additional intravitreal RBZ-BS injections were administered.

### 2.4. Clinical Evaluations

The outcome measures in this study were change in BCVA and central macular thickness (CMT) from baseline every 3 months up to 12 months. For the decimal, visual acuities were converted to logarithmic minimum angle of resolution (logMAR) values for the analysis of BCVA. The rate of dry macula was also evaluated. The total number of IVRBS treatments, the number of treatments administered to achieve dry macula, and the dry macula rate at each observation period were also examined. Severe complications, including intraocular inflammation, infectious endophthalmitis, rhegmatogenous retinal detachment, cerebral infarction, and myocardial infarction, were also investigated.

### 2.5. Statistical Analysis

The Wilcoxon signed-rank-sum test was used to compare BCVA and CMT before and after treatment. The Mann–Whitney U test was used to compare changes in BCVA, CMT, CNV area, and number of treatments between the naïve and previously treated group. Fisher’s exact test was used to compare the rate of dry macula between the two groups. The Benjamini–Hochberg method was used to correct the *p* value by controlling the false-discovery rate. IBM SPSS Statistics 24.0 (IBM Japan, Ltd., Tokyo, Japan) was used for statistical analysis, in which *p* values < 0.05 were regarded as significant.

## 3. Results

During the study period, 24 sets of eyes of 24 patients received RBZ-BS, of which 3 sets of eyes (12.5%) did not complete the 1-year follow-up and were excluded. Therefore, 21 sets of eyes of 21 patients with mCNV were included in this study. Patients in the study had a mean age of 69.0 ± 11.8 years (range: 50–86 years), with 4 males and 17 females. All patients met the diagnostic criteria for high myopia, with a mean axial length of 29.48 ± 2.26 mm and a mean equivalent spherical power of −10.29 ± 8.24 D. Seven (33.3%) of the sets of eyes had intraocular lenses. Twelve sets of eyes (57.1%) were in the naïve group and nine sets of eyes (42.9%) were in the previously treated group. The CNV area was 0.92 ± 0.66 mm^2^ in the naive group and 0.50 ± 0.34 mm^2^ in the previously treated group, with no significant difference between the two groups (*p* = 0.10). In the previously treated group, the CNV area at initial treatment was 0.40 ± 0.31 mm^2^, which was significantly smaller (*p* < 0.05) than at baseline. Eight sets of eyes (38.1%) were treated with ranibizumab and 1 set of eyes (4.8%) with aflibercept; the mean number of doses was 2.6 ± 1.8 (range: 1–6), and the mean time to start BBZ-BS was 31.5 ± 42.3 months (range: 5.4–128.9 months). Eight sets of eyes were treated with PRN and 1 set of eyes with the treat and extend (TAE) method (Table 1). No patients were switched to other anti-VEGF drugs during the study period. Other baseline information and data during the study were included in the Supplementary File.

**Figure 1:**
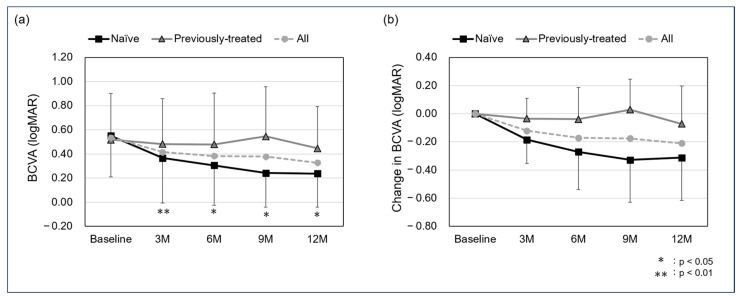
Mean best corrected visual acuity (BCVA) (**a**) and change in BCVA (**b**) from baseline to 12 months (logMAR). A significant difference was seen between baseline and the 3-, 6-, 9-, and 12-month follow-up in the naïve group (*p*: <0.01, <0.05, <0.05, and <0.05, respectively) but not seen in the previously treated group (*p*: 0.93, 0.67, 1.00, and 1.00, respectively). The mean change in BCVA in the naïve and previously treated group was −0.18 ± 0.17 and −0.04 ± 0.15 at the 3-month follow-up, −0.27 ± 0.27 and −0.04 ± 0.23 at 6 months, −0.33 ± 0.30 and 0.03 ± 0.22 at 9 months, and −0.31 ± 0.30 and −0.97 ± 0.27 at 12 months, with no significant difference in all follow-up periods (*p*: 0.11, 0.08, 0.06, and 0.11, respectively).

The mean BCVA in all cases was 0.54 ± 0.35 at baseline, 0.42 ± 0.37 at the 3-month follow-up, 0.38 ± 0.38 at 6 months, 0.38 ± 0.7 at 9 months, and 0.33 ± 0.32 at 12 months. For the naïve and previously treated groups, the mean BCVA was 0.55 ± 0.34 and 0.52 ± 0.38 at baseline, 0.37 ± 0.37 and 0.48 ± 0.38 at the 3-month follow-up, 0.30 ± 0.33 and 0.48 ± 0.43 at 6 months, 0.24 ± 0.55 and 0.55 ± 0.41 at 9 months, and 0.24 ± 0.28 and 0.45 ± 0.35 at 12 months. A significant difference was observed in the naïve group between baseline and the 3-, 6-, 9-, and 12-month follow-ups (*p*: <0.01, <0.05, <0.05, and <0.05, respectively), while no significant differences were found in the previously treated group (*p*: 0.93, 0.67, 1.00, and 1.00, respectively). In all cases, the mean change in BCVA was −0.12 ± 0.17 at the 3-month follow-up, −0.17 ± 0.27 at 6 months, −0.18 ± 0.32 at 9 months, and −0.21 ± 0.31 at 12 months. The mean change in BCVA for the naïve and previously treated group was −0.18 ± 0.17 and −0.04 ± 0.15 at the 3-month follow-up, −0.27 ± 0.27 and −0.04 ± 0.23 at 6 months, −0.33 ± 0.30 and 0.03 ± 0.22 at 9 months, and −0.31 ± 0.30 and −0.97 ± 0.27 at 12-months, with no significant difference in all follow-up periods (*p*: 0.11, 0.08, 0.06, and 0.11, respectively) (Figure 1).

**Figure 2:**
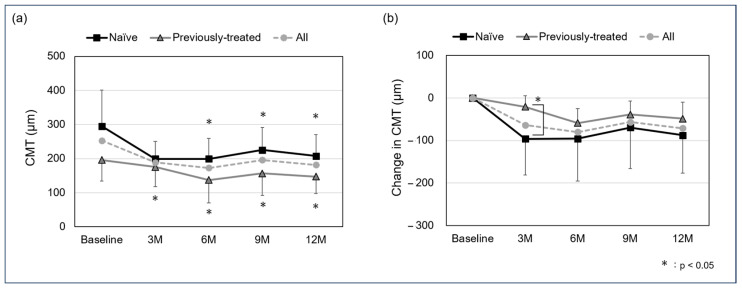
Mean central macular thickness (CMT) (**a**) and change in CMT (**b**) from baseline to 12 months (logMAR). A significant difference was seen between baseline and the 3-, 6-, 9-, and 12-month follow-up in the naïve group (*p*: <0.05, <0.05, <0.05, and <0.05, respectively) and between the 6-, 9-, and 12-month follow-up in the previously treated group (*p*: 0.0502, <0.05, <0.05, and <0.05, respectively). The mean change in CMT in the naïve and previously treated group was −96.3 ± 84.9 µm and −20.8 ± 25.9 µm at the 3-month follow-up, −95.8 ± 99.6 µm and −59.1 ± 33.7 µm at 6 months, −69.8 ± 95.8 µm and −39.2 ± 31.7 µm at 9 months, and −87.9 ± 89.0 µm and −48.9 ± 39.1 µm at 12 months, with a significant difference in the 3-month follow up (*p*: <0.05, 0.62, 0.23, and 0.36, respectively).

The mean CMT in all cases was 252.8 ± 100.8 µm at baseline, 188.9 ± 54.3 µm at the 3-month follow-up, 172.7 ± 69.3 at 6 months, 196.1 ± 72.5 µm at 9 months, and 181.6 ± 64.1 µm at 12 months. For the naïve and previously treated group, the mean CMT was 295.3 ± 105.2 µm and 196.1 ± 62.0 µm at baseline, 199.0 ± 51.7 µm and 175.3 ± 57.6 µm at the 3-month follow-up, 199.4 ± 60.2 µm and 137.0 ± 67.2 µm at 6 months, 225.5 ± 65.7 µm and 156.9 ± 64.7 µm at 9 months, and 207.3 ± 63.0 µm and 147.2 ± 50.1 µm at 12 months. A significant difference was observed in the naïve group between baseline and the 3-, 6-, 9-, and 12-month follow-ups (*p*: <0.05, <0.05, <0.05, and <0.05, respectively), and in the previously treated group between the 6-, 9-, and 12-month follow-ups (*p*: 0.0502, <0.05, and <0.05, respectively). In all cases, the mean change in CMT was −63.9 ± 75.5 µm at the 3-month follow-up, −80.1 ± 79.1 at 6 months, −56.7 ± 75.4 µm at 9 months, and −71.2 ± 73.2 µm at 12 months. For the naïve and previously treated groups, mean change in CMT was −96.3 ± 84.9 µm and −20.8 ± 25.9 µm at the 3-month follow-up, −95.8 ± 99.6 µm and −59.1 ± 33.7 µm at 6 months, −69.8 ± 95.8 µm and −39.2 ± 31.7 µm at 9 months, and −87.9 ± 89.0 µm and −48.9 ± 39.1 µm at 12 months, with a significant difference in the 3-month-follow up (*p*: <0.05, 0.62, 0.23, and 0.36, respectively) (Figure 2).

**Figure 3:**
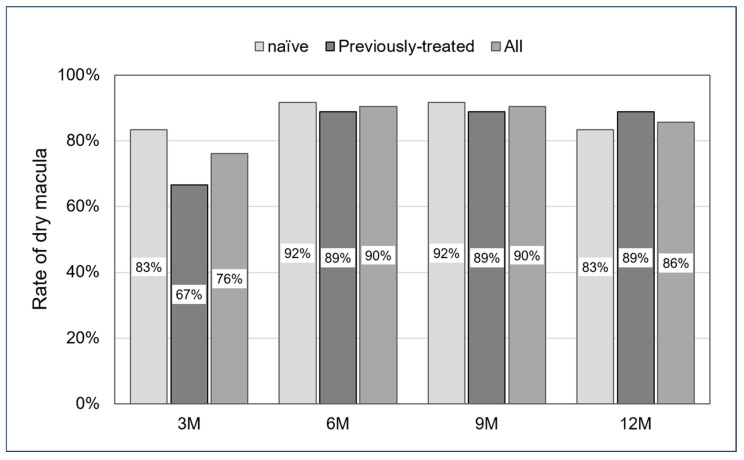
Rate of dry macula from 3 to 12 months. No significant differences were seen in all follow-up periods (*p*: 0.35, 0.69, 0.69, and 0.61, respectively).

In all cases, the dry macula rate was 76% at the 3-month follow-up, 90% at 6 months, 90% at 9 months, and 86% at 12 months. For the naïve and previously treated groups, the dry macula rate was 83% and 67% at the 3-month follow-up, 92% and 89% at 6 months, 92% and 89% at 9 months, and 83% and 89% at 12 months. No significant differences were observed across all follow-up periods (*p*: 0.35, 0.69, 0.69, and 0.61, respectively) (Figure 3).

The mean number of IVRBS required to achieve a dry macula in all cases was 1.6 ± 0.6 (range: 1–3), and during the 12-month period, it was 3.1 ± 1.7 (range: 1–7). In the naïve group, the mean number of IVRBS to achieve a dry macula was 1.7 ± 0.7 (range: 1–3), and 1.4 ± 0.5 (range: 1–2) in the previously treated group. During the 12-month period, the mean number was 3.1 ± 1.7 (range: 1–7) in the naïve group and 3.3 ± 1.3 (range: 1–5) in the previously treated group, with no significant difference between the two groups (*p*: 0.45 and 0.42, respectively) (Table 2).

During this study, no AEs developed, such as cerebral infarction, myocardial infarction, or other systemic disease, or intraocular inflammation, hemorrhage, or other event attributable to IVRBS treatment.

## 4. Discussion

This is the first clinical report of intravitreal injection of RBZ-BS for mCNV. The results of this study demonstrate that intravitreal injection of RBZ-BS is effective in treating mCNV in Japanese patients over a one-year period. The primary outcome measures, including BCVA and CMT, showed significant improvements in the naïve group, indicating the efficacy of RBZ-BS in managing mCNV.

The mean BCVA in the naïve group improved significantly from 0.55 ± 0.34 at baseline to 0.24 ± 0.28 at the 12-month follow-up. This improvement suggests that RBZ-BS is effective in restoring vision in naïve patients. In previous reports, the original product, ranibizumab, produced rapid improvements in mean BCVA from baseline to month 3. In addition, continuation of ranibizumab treatment to month 12 maintained the improvement, resulting in an increase in BCVA of approximately 14 letters [12]. This study’s findings are consistent with previous studies on the efficacy of the original ranibizumab in treating mCNV. In contrast, the previously treated group did not show a statistically significant improvement in BCVA over the same period. This may be attributed to the fact that these patients had already received treatments with other anti-VEGF agents, such as original ranibizumab or aflibercept, prior to switching to RBZ-BS. Also, the fact that the mean time to IVRBS induction was 31.5 months may suggest that the treatment effect reached a plateau before the introduction of RBZ-BS. In the previously treated group, visual acuity was 0.40 ± 0.31 at the initial treatment and 0.50 ± 0.34 at baseline (RBZ-BS administration), which was significantly worse, and it seems clear that the effects of RBZ-BS introduction from this point on would be limited (please refer to the Supplementary File for visual acuity at the initial treatment).

A significant reduction in CMT was observed in both the naïve and the previously treated groups. In the naïve group, the mean CMT decreased from 295.3 ± 105.2 µm at baseline to 207.3 ± 63.0 µm at the 12-month follow-up (*p* < 0.05). Similarly, the previously treated group showed a reduction from 196.1 ± 62.0 µm to 147.2 ± 50.1 µm (*p* < 0.05). CMT in patients with mCNV has previously shown improvement with intravitreal injection of anti-VEGF drugs: In the IVA (intravitreal aflibercept) group, it significantly decreased from 384.3 ± 119.1 μm at baseline to 305.9 ± 75.4 μm at 12 months (*p* < 0.001). At 12 months after IVR therapy, mean CMT significantly decreased from 366.5 ± 102.3 μm at baseline to 323.6 ± 103.6 μm [24]. In another report, the mean CMT significantly improved from baseline 384.3 ± 119.1 μm to 305.9 ± 75.4 μm (*p*: 0.02) [25]. In a study comparing ranibizumab with the number of times during the loading phase, there was a significant decrease in both groups, from 244.5 µm to 189.3 µm in the 1 + PRN group and from 262.9 µm to 197.6 µm in the 3 + PRN group [26]. The number of treatments per year was 2.04 ± 1.22 in the 1 + PRN group and 3.58 ± 0.72 in the 3 + PRN group, with the significantly lower number in the 1 + PRN group, indicating that one treatment in the induction period is sufficient to produce a therapeutic effect and may control excessive treatment. Risk factors for re-treatment in this study, however, included 1 + PRN, women, older age, and thicker retina, suggesting that caution should be exercised in the case of 1 + PRN, which may be more prone to recurrence. These reductions in CMT align with the findings of previous studies that demonstrated the efficacy of anti-VEGF treatments in reducing macular edema and improving retinal morphology [24,25,26]. The comparable effectiveness of RBZ-BS in reducing CMT underscores its potential as a cost-effective alternative to the original ranibizumab.

The rate of achieving dry macula, defined as the absence of all intraretinal and subretinal fluid and intraretinal and subretinal hemorrhages, was high in both groups. The naïve group achieved a dry macula rate of 83% at 3 months and 83% at 12 months, while the previously treated group achieved 67% at 3 months and 89% at 12 months. Cohen et al. reported that 29 patients with mCNV were treated with intravitreal ranibizumab and showed complete regression of CNV in 13 sets of eyes at 6 months [27]. Also, Bruyerre et al. detected subretinal hyperreflective exudates resolved completely in 29 of 31 sets of eyes (93.5%) and partially in 2 of 31 sets of eyes (6.5%) [28]. Similar outcomes were detected by Niccolò Castellino et al., with the dry macular ratio improving significantly from 41% to 4.9% after 12 months compared to baseline in an immature group with anti-VEGF injections [29]. We believe that the results of this study are comparable to previous reports examining morphologic changes after treatment with ranibizumab. The worse 3-month dry macula rate in the previously treated group, although not significantly different, may be because some time had passed since the initial treatment and morphological improvement was delayed, as mentioned in the previous discussion of BCVA.

While the efficacy of RBZ-BS in this study aligns with that of the original ranibizumab, it is essential to consider the economic implications of using biosimilars. The high cost of original biologic drugs like ranibizumab poses a significant financial burden on patients and healthcare systems [30]. A. Sharma et al. reported the possibility that anti-VEGF biosimilars have the potential to reduce costs by up to 30% [31]. The introduction of biosimilars offers a viable solution to this problem by providing similar therapeutic outcomes at a lower cost. The use of RBZ-BS can potentially reduce treatment costs while maintaining high efficacy and safety, thereby improving accessibility and adherence to treatment for patients with mCNV. The safety profile of RBZ-BS in this study was favorable, with no adverse events (AEs) reported, such as cerebral infarction, myocardial infarction, intraocular inflammation, or hemorrhage. This finding is consistent with previous studies that reported a low incidence of AEs with the original ranibizumab [9,12]. The absence of significant AEs in this study reinforces the safety of RBZ-BS, making it a reliable option for long-term management of mCNV.

This study has several limitations. First, the retrospective design may introduce selection bias, and the relatively small sample size limits the generalizability of the findings. Second, the follow-up period was limited to one year, and longer-term outcomes are needed to fully assess the efficacy and safety of RBZ-BS. Future prospective, randomized controlled trials with larger sample sizes and longer follow-up periods are warranted to confirm these findings and establish the long-term benefits and safety of RBZ-BS in treating mCNV. Additionally, further studies should explore the cost-effectiveness of RBZ-BS in comparison to other anti-VEGF agents. As healthcare systems worldwide grapple with rising medical costs, the economic evaluation of biosimilars will play a crucial role in decision-making processes for treatment guidelines and reimbursement policies.

## 5. Conclusions

In conclusion, the one-year outcomes of intravitreal injection of ranibizumab biosimilar for myopic choroidal neovascularization in Japanese patients demonstrate significant improvements in BCVA and reductions in CMT, particularly in treatment-naïve patients. The high rates of achieving dry macula and the favorable safety profile further support the use of RBZ-BS as an effective and safe alternative to the original ranibizumab. The introduction of RBZ-BS holds promise for reducing the financial burden on patients and healthcare systems while maintaining high therapeutic efficacy, making it a valuable addition to the treatment arsenal for mCNV.

## Figures and Tables

**Table 1:** Patient characteristics at baseline.

Characteristics	Mean ± SD or No. (%)
No. eyes	21
Age, years	69.0 ± 11.8
Gender (male), eyes	4 (19.0)
Intraocular lens, eyes	7 (33.3)
Axial length, mm	29.48 ± 2.26
History of treatment	
Naïve	12 (57.1)
Previously treated	9 (42.9)
Intravitreal ranibizumab	8 (38.1)
Intravitreal aflibercept	1 (4.8)
Best corrected visual acuity, logMAR	0.54 ± 0.35
Central macular thickness, µm	252.8 ± 100.8
CNV area, mm^2^	0.74 ± 0.58

**Table 2:** Number of treatments.

Mean Number (Range)	All	Naïve Group	Previously Treated Group	*p* Value
Up to dry macula	1.6 ± 0.6 (1–3)	1.7 ± 0.7 (1–3)	1.4 ± 0.5 (1–2)	0.45
Total	3.2 ± 1.5 (1–7)	3.1 ± 1.7 (1–7)	3.3 ± 1.3 (1–5)	0.42

## Data Availability

The data presented in this study are available in the Supplementary Materials.

## Supplementary Materials

The following supporting information can be downloaded at: https://www.mdpi.com/article/10.3390/jcm13164641/s1, Supplementary.xlsx.
